# Serum CA19-9 and CEA levels, serum CAT, GSH, oxidised glutathione levels, 8-dihidro-2'-deoksiguanosina and F2-isoprostanes levels in colorectal cancer patients and Lactobacillus: A randomised double-blind controlled trial

**DOI:** 10.5937/jomb0-56528

**Published:** 2025-10-28

**Authors:** Shiru Chen, Weili Ning, Jiye Zhang, Zhenting Wu, Hang Zhou, Ying Liu

**Affiliations:** 1 The First Affiliated Hospital of Heilongjiang University of Traditional Chinese Medicine, Department of Digestive Minimally Invasive Diagnosis and Treatment, Harbin, Heilongjiang 150000, China; 2 Heilongjiang University of Traditional Chinese Medicine, Harbin, Heilongjiang 150000, China

**Keywords:** Lactobacillus, colorectal cancer, oxidative stress, p38 MAPK, JNK, NRF2, Lactobacillus, kolorektalni karcinom, oksidativni stres, p38 MAPK, JNK, NRF2

## Abstract

**Background:**

Oxidative stress (OS) plays a crucial role in colorectal cancer (CRC) progression. Lactobacillus has been proposed as a potential modulator of OS. This randomised controlled trial aimed to evaluate the effects of Lactobacillus supplementation on OS markers and its related signalling pathways in CRC patients after surgery.

**Methods:**

A total of 76 CRC patients were enrolled and randomised into two groups: the study group (n=39) received Lactobacillus supplementation, while the control group (n=37) received a placebo. The intervention lasted for six months following surgery. Serum levels of catalase (CAT), glutathione (GSH), oxidised glutathione (GSSG), 8-oxo-7,8-dihydro-2'-deoxyguanosine (8-oxodG), and F2-isoprostanes (F2-IsoPs) were measured. In addition, the nuclear factor erythroid 2-related factor 2/Kelch-like ECH-associated protein 1 (NRF2/KEAP1), p38 mitogen-activated protein kinase (MAPK), and c-Jun N-terminal kinase (JNK) signalling pathways were assessed via western blot analysis.

**Results:**

Following Lactobacillus supplementation, serum carcinoembryonic antigen (CEA) levels significantly decreased, whereas carbohydrate antigen 19-9 (CA19-9) levels remained unchanged. OS marker analysis demonstrated increased CAT, GSH, and F2-IsoPs levels and decreased GSSG and 8-oxodG levels in the study group compared to the control group. Western blot results revealed that NRF2, ASK1, MKK3, p-p38, and MKK4 protein levels were significantly reduced after Lactobacillus intervention, while KEAP1 and p-JNK remained unchanged.

**Conclusions:**

Oral administration of Lactobacillus for six months reduced OS marker levels and inhibited NRF2/KEAP1, p38 MAPK, and JNK signalling pathways in CRC patients after surgery. These findings suggest that Lactobacillus may contribute to CRC management by modulating oxidative stress.

## Abstract (sr)

**Metode:**

Ukupno 76 pacijenata sa CRC-om je nasumično podeljeno u dve grupe: studijsku (n=39), koja je dobijala Lactobacillus, i kontrolnu (n=37), koja je dobijala placebo. Intervencija je trajala šest meseci nakon operacije. Mereni su serumski nivoi katalaze (CAT), glutationa (GSH), oksidovanog glutationa (GSSG), 8-oxo-7,8-dihidro-2'-deoksigvanozina (8-oxodG) i F2-izoprostana (F2-IsoPs). Takođe su analizirani signalni putevi povezani sa NRF2/KEAP1, p38 MAPK i c-Jun N-terminalnom kinazom (JNK) primenom "western blot metode".

**Rezultati:**

Nakon suplementacije Lactobacillus-om, nivo karcino embrionalnog antigena (CEA) u serumu bio je značajno smanjen, dok se nivo karbohidratnog antigena 19-9 (CA19-9) nije menjao. Analiza markera OS je pokazala porast nivoa CAT, GSH i F2-IsoPs, te smanjenje nivoa GSSG i 8-oxodG u studijskoj grupi u poređenju sa kontrolnom. Western blot analiza otkrila je značajno smanjenje nivoa proteina NRF2, ASK1, MKK3, p-p38 i MKK4 nakon intervencije, dok nivoi KEAP1 i p-JNK nisu pokazali značajne promene.

**Zaključak:**

Oralna suplementacija Lactobacillus-om tokom šest meseci smanjuje markere oksidativnog stresa i inhibira NRF2/KEAP1, p38 MAPK i JNK signalne puteve kod pacijenata sa CRC-om nakon operacije. Ovi nalazi ukazuju na potencijalnu ulogu Lactobacillus-a u upravljanju kolorektalnim karcinomom kroz modulaciju oksidativnog stresa.

## Introduction

Colorectal cancer (CRC) is the second most common cause of cancer death in the United States.According to colorectal cancer statistics, in 2023, 153,020 people will be diagnosed with CRC, and52,550 cases will die from CRC [1]. The aetiology of CRC is related to hereditary susceptibility syndrome, family history of CRC, inflammatory bowel disease, especially obesity and dietary risk factors [2]. The main clinical symptoms of CRC are hematochezia, changes in defecation habits and changes in stool consistency [3]. Certain foods (such as red meat, high-fat and low-fiber foods) may increase the risk of CRC [4]. These environmental and life factors may have an impact on the inherent intestinal flora, resulting in intestinal flora imbalance.

Therefore, considering the influence of dietary habits on immune response caused by intestinal bacteria, the composition of intestinal flora and intestinal homeostasis, intestinal flora or its derived metabolites may be direct environmental regulators that lead to CRC.

The gastrointestinal tract contains as many bacteria as cells that constitute the human body [5]. More and more studies reveal that the gut microbiota regulates the efficacy and toxicity of cancer therapy [6]. Intestinal flora can participate in the metabolism of substances in the human body and provide important substances to the body, such as butyrate-producing bacteria, which can convert organic acids into short-chain fatty acids (SCFA) that are beneficial to the host. SCFAs provide energy for colon cells, enhance intestinal barrier function and reduce oxidative stress response. *Lactobacillus *is one of the most commercialised lactic acid probiotics, and numerous studies have confirmed their advantages in preventing and treating CRC [7] [8] [9].

Oxidative stress (OS) is caused by an imbalance between pro-oxidant molecules and the cell’s anti-oxidant capacity [10]. Glutathione (GSH) is one of the most important anti-oxidants in cells and participates in the regulation of oxidative stress by directly eliminating reactive oxygen species (ROS) or as a cofactor of glutathione peroxidase (GPx) [11]. In CRC, the changes in GSH levels are closely related to the occurrence and development of tumours [12]. Catalase (CAT) is a key enzyme for decomposing hydrogen peroxide (H_2_O_2_) within cells [13]. By catalysing the decomposition of H_2_O_2_ into water and oxygen, it prevents oxidative damage caused by the accumulation of H_2_O_2_ [14]. In CRC, the expression and activity changes of CAT may affect the microenvironment and progression of the tumour [15]. Oxidised glutathione (GSSG) is the oxidised form of GSH and is produced during intracellular oxidative stress [16]. The changes in GSSG in CRC reflect the intracellular REDOX imbalance and can participate in the occurrence and development of CRC by promoting oxidative stress [17]. 8-oxo-7,8-dihydro-2’-deoxyguano sine (8-oxodG) is an oxidative admixture formed by ROS attacking the carbon atom at the 8th position of the guanine base in DNA molecules, and it is a marker of DNA oxidative damage [18]. Studies have shown that the level of 8-oxodG in the serum or tissues of patients with CRC is associated with tumour stage, lymph node metastasis and poor prognosis [19]. F2-isoprostanes (F2-IsoPs) are the oxidation product of arachidonic acid under non-enzymatic free radical catalysis [20]. It is a biomarker of lipid peroxidation and reflects the level of oxidative stress in the body [21]. Among patients with CRC, the level of F2-IsoPs is significantly increased, reflecting an increase in the level of oxidative stress in the body [22]. ROS and reactive nitrogen species can cause inflammation, which contributes to tumour development [23]. The body made an inflammatory response to OS-stimulated damage of intestinal mucosa. Repeated inflammation causes chronic inflammation and induces autoimmune processes [24]. Cell integrity was then destroyed, and defensive function in the gut mucosa was damaged, which subsequently led to mucosal injury and pathogen invasion [25]. Inflammation precedes or accompanies tumour development in some cases [26]. ROS are produced primarily by macrophages and neutrophils, and they can also be produced by certain microorganisms [27]. *Lactobacillus *has the potential to reduce OS to prevent disease [28]. In addition, carcinoembryonic antigen (CEA) and carbohydrate antigen 19-9 (CA19-9) are typical tumour markers that can be used for the diagnosis of CRC and show abnormally high expression levels in CRC patients [29]. In this study, we evaluated the influences of oral supplementation with *Lactobacillus *on OS markers and on signaling pathways in response to OS in patients with CRC after surgery.

## Materials and methods

### Participants

**Figure 1:**
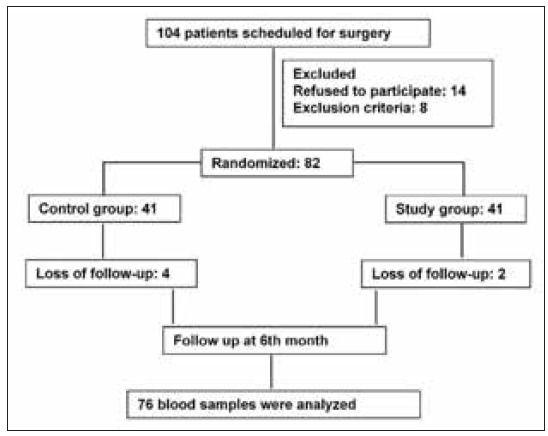
Flow Chart.

From May 2023 to August 2024, a randomised, double-blind, controlled trial was conducted at The First Affiliated Hospital of Heilongjiang University of Traditional Chinese Medicine. Both patients and the data analyser were blind to the design and grouping of this study. The Ethics Committee of the First Affiliated Hospital of Heilongjiang University of Traditional Chinese Medicine approved this trial. All patients were told the purpose of this study and signed the written informed consent. A total of 104 patients met the inclusion criteria, and 22 patients were excluded. A flowchart of the schedule of this study is shown in Figure 1.

The inclusion criteria were: (1) patients were aged 20–75 years old, diagnosed with CRC, and need surgical resection; (2) CRC was conformed pathohistologically by two experts. The exclusion criteria were: (1) patients had distant metastases; (2) patients had used antibiotics or consumed pro/pre/syn-biotic products within two weeks; (3) patients had severe disorders in lung, liver, heart, and kidneys; (4) patients had a history of colorectal adenoma or inflammatory bowel disease; (5) patients were pregnant or had other tumours.

### Treatments

*Lactobacillus *complex tablets consisting of live* Lactobacillus lactis* (>70 CFU), *Lactobacillus acidophilus* (>7000 CFU), and *Streptococcus lacti*s (>1.4×10^4^ CFU) were manufactured by Jiangsu Mei tong Pharmaceutical Co., LTD (batch number: H19980184; Taizhou, Jiangsu, China). Each tablet weighed 0.33 g and contained over 2 × 10^4^ CFU of total *Lactobacillus*. Placebo tablets contain starch and have a taste and texture similar to *Lactobacillus *complex tablets. All tablets were kept at 2–8°C, avoiding light.

The nurses instructed patients in the study group to consume *Lactobacillus *complex tablets and the patients in the placebo group to consume control tablets after surgery for four weeks. Patients take the products orally after a meal in the morning and evening for six months.

### Collection of blood sample

Five mL of blood was taken from all participants before surgery and in the 6^th^ month after intervention. Blood was transferred into BD vacutainer and let stand for 30 min for clotting. It was then centrifuged for 15 min at 1000 × g, and the upper layer, namely serum, was stored at -80°C till use.

### Determination of tumour markers

Preoperative serum CA19-9 and CEA levels are recommended to be routinely measured in patients with CRC [30]. Analysis of serum CA19-9 and CEA was conducted using an ARCHITECT c4000 clinical chemistry analyser (Abbott, Chicago, IL, USA) before surgery and after intervention for 6 months.

### Determination of OS indexes

Serum CAT, GSH, oxidised glutathione (GSSG) levels and urine 8-oxo-7,8-dihidro-2’-deoxiguanosina (8-oxodG) and F2-Isoprostanes (F2-IsoPs) levels were analysed. Except for 8-oxodG that was detected by High-Performance Chromatography with Electrochemical Detection (19), other OS markers including CAT (#ab83464, Abcam, Shanghai, China), GSH (#S0053, Beyotime, Shanghai, China), GSSG (#S0053, Beyotime, Shanghai, China), and F2-IsoPs (#ab175819, Abcam) were determined using corresponding commercial kits.

### Western blot analysis

To remove high-abundant protein and decrease sample complexity, serum samples were treated with a ProteoExtract® kit (#122643, Millipore, USA). Next, protein samples were subjected to a routine western blotting procedure [31]. Equal amounts (4 μg) of the proteins were electrophoresed on 15% SDS-PAGE and transferred to Immobilon membranes (Millipore, Bedford, MA). Then, the membranes were incubated with primary antibodies, including anti-NRF2 (1/500, #ab62352, Abcam), p-NRF2 (1/5000, #ab76026, Abcam), KEAP1 (1/2000, #ab227828, Abcam), ASK1 (1/1000, #ab45178, Abcam), MKK3 (1/5000, #ab195037, Abcam), p38 (1/1000, #abab182453, Abcam), p-p38 (1/1000, #ab178867, Abcam), MKK4 (1/1000, #ab33912, Abcam), JNK (1/1000, #ab110724, Abcam), p-JNK (1/2000, #ab307802, Abcam), and GAPDH (1/500, #ab8245, Abcam). After incubation overnight at 4°C, the membrane was incubated with secondary antibody anti-IgG. The Immobilon Western chemiluminescent horseradish peroxidase substrate (Millipore) was used for visualising the labelling, and a LAS-3000 mini system (Fujifilm, Tokyo, Japan) was used to observe the signals.

### Statistical analysis

Data were analysed using the Prism software version 8.0. For clinicopathological data, except for age, which was expressed as means ± SD, other data were expressed as n (%), and a t-test was conducted for comparison of age. At the same time, the χ^2^ test was applied for comparison with other clinicopathological data. The measured cytokine level was ex pressed as means ± SD and analysed using analysis of variance. P<0.05 was used to indicate statistical significance.

## Results

### Clinical characteristics of CRC patients

Clinicopathological characteristics, including gender, age, history of CRC, history of smoking, comorbidities, TNM stage, differentiation, tumour location, and adjuvant therapy of patients in two groups, were identified (Table 1). Major patients were at TNM stage II–III, had well/moderately differentiated tumours, and tumours were primarily located right. There was no statistical difference in these characteristics between the two groups. These data suggested that patients were homogeneous between the two groups.

**Table 1:** Clinicopathological characteristics of patients in two groups. TNM: tumour/node/metastasis.

Parameters		Study (n=39)	Control (n=37)	t/χ^2^	P
Age (years)		64.21±8.12	62.58±7.19	0.92	0.35
Gender	Male	27 (69.23%)	23 (62.16%)	0.42	0.51
	Female	12 (30.77%)	14 (37.84%)		
History of CRC	Yes	13 (33.33%)	10 (27.03%)	0.35	0.54
	No	26 (66.67%)	27 (27.03%)		
History of smoking	Yes	21 (53.85%)	22 (72.97%)	0.24	0.62
	No	18 (46.15%)	15 (40.54%)		
Comorbidities	Hypertension	21 (53.85%)	19 (51.35%)	0.04	0.82
	Dyslipidaemia	17 (43.59%)	18 (48.65%)	0.19	0.65
	Diabetes mellitus	3 (7.69%)	2 (5.41%)	0.16	0.68
	Chronic kidney disease	2 (5.13%)	4 (10.81%)	0.84	0.35
	Chronic heart disease	3 (7.69%)	1 (2.70%)	0.94	0.33
TNM Stage	I	4 (10.26%)	6 (16.22%)	0.69	0.87
	II	13 (33.33%)	12 (32.43%)		
	III	16 (41.03%)	13 (35.13%)		
	IV	6 (15.38%)	6 (16.22%)		
Differentiation	Well-differentiated	18 (46.15%)	15 (40.54%)	0.52	0.76
	Moderately differentiated	18 (46.15%)	20 (54.05%)		
	Poorly differentiated	3 (7.69%)	2 (5.41%)		
Location	Right	15 (38.46%)	14 (37.84%)	0.00	0.95
	Left	24 (61.54%)	23 (62.16%)		
Adjuvant therapy	Yes	12 (30.77%)	8 (21.62%)	0.81	0.36
	No	27 (69.23%)	29 (78.38%)		

### Comparison of tumour markers

**Figure 2:**
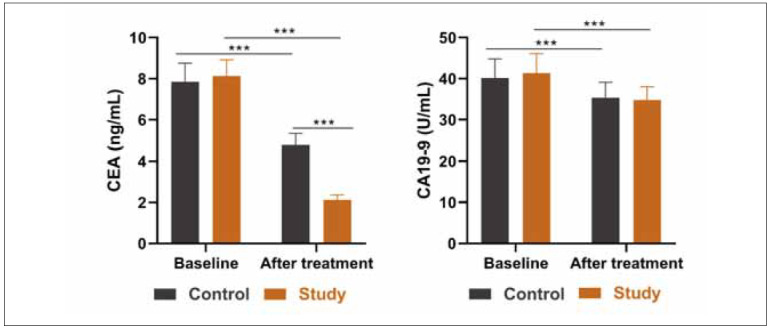
Serum CEA and CA19-9 levels in two groups.<br>***p<0.001. Data were analysed using analysis of variance.

There was no significant difference in baseline CEA and CA19-9 levels between the two groups. After treatment, both CEA and CA19-9 levels were decreased. It was observed that the probiotics group had a lower CEA level than the placebo group, while CA19-9 levels showed no significant difference after treatment (Figure 2).

### Comparison of OS markers

**Figure 3:**
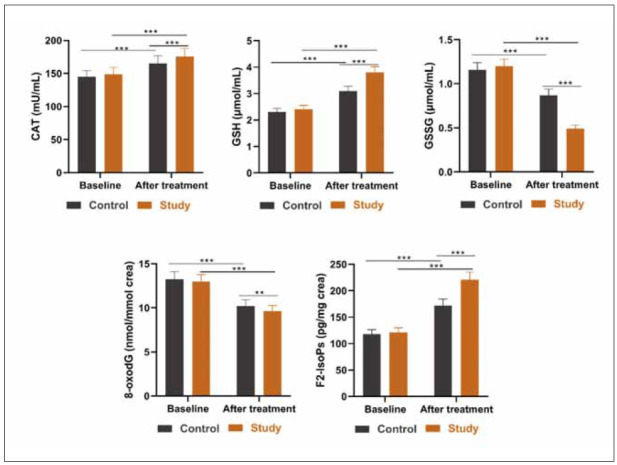
Serum and urine levels of OS markers in two groups.<br>CAT: catalase, GSH: glutathione, GSSG: oxidised glutathione, 8-oxodG: 8-oxo-7,8-dihidro-2’-deoxiguanosin, F2-IsoPs: F2-Isoprostanes. **p<0.01, ***p<0.001. Data were analysed using analysis of variance.

Next, OS markers, including CAT, GSH, GSSG, 8-oxodG, and F2-IsoPs, were evaluated between the two groups. There was no significant difference in their baseline levels between the two groups. After treatment, CAT, GSH, and F2-IsoPs levels were enhanced, and GSSG and 8-oxodG levels weredecreased. The probiotics group showed higher CAT, GSH, and F2-IsoPs levels and lower GSSG and 8-oxodG levels than the placebo group after treatment (Figure 3).

### Comparison of OS-related pathways

**Figure 4:**
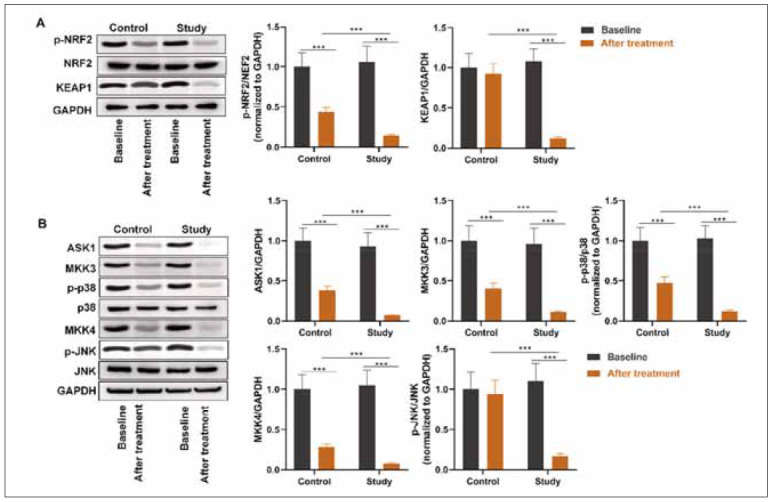
OS-related pathways in two groups.<br>(A) Proteins of the NRF2 pathway, including p-NRF2, NRF2, and KEAP1, were assessed by western blotting. (B) Proteins of the p38 MAPK pathway, including ASK1, MKK3, p-p38, p38 and those of the JNK MAPK pathway, including MKK4, p-JNK, and JNK, were assessed by western blotting. ***p<0.001. Data were analysed using analysis of variance.

Subsequently, the NRF2 and ASK1/p38/JNK pathways were assessed. Decreased protein levels of NRF2, ASK1, MKK3, p-p38, and MKK4 after treatment were observed. There were no significant differences in KEAP1 and p-JNK levels. The study group exhibited lower protein levels of NRF2, KEAP1, ASK1, MKK3, p-p38, MKK4, and p-JNK than control group after intervention. All these results suggested that *Lactobacillus *had a suppressive effect on the NRF2 and ASK1/p38/JNK pathways (Figure 4).

## Discussion

*Lactobacillus* is widely reported to confer beneficial effects on human health, and more and more studies have focused on the association of *Lactobacillus *and cancers, especially CRC. The metabolite of *Lactobacillus plantarum*, indole-3-lactic acid, can transcriptionally suppress Saa3 expression to epigenetically regulate CD8+ T cell immunity epigenetically, thus ameliorating CRC tumourigenesis [32]. *Lactobacillus gallinarum*-derived metabolites have anti-PD1 efficacy by modulating the IDO1/Kyn/AHR pathway to suppress regulatory T cells in CRC [33]. *Lactobacillus gasseri* 505 reduces the incidence of colonic tumours and damage to the colonic mucosa effectively in azoxymethane/dextran sodium sulfate-induced mouse model of colitis-associated CRC [34]. All these findings reveal that *Lactobacillus *confers anticancer effects. Our study held the same opinion that *Lactobacillus *was conducive to defending the body against CRC. In our clinical trial, tumour marker CEA was suppressed by oral consummation of *Lactobacillus *for six months after surgery. Consistently, Agah et al. suggested that *Lactobacillus acidophilus* could reduce the serum levels of CEA and CA19-9 tumour markers in mouse colon cancer [35]. However, *Lactobacillus *may be harmful in specific cases. For example, tumour-resident *Lactobacillus *iners is associated with decreased survival in patients with cancer, induces resistance of tumour cells to chemotherapy and radiation, and leads to metabolic reprogramming [36]. The roles of *Lactobacillus *double-edged sword in cancers are closely associated with the source and species of *Lactobacillus*.

Moreover, we found that circulating OS markers of CAT, GSH, GSSG, 8-oxodG, and F2-IsoPs were decreased in CRC patients after surgery. The intervention of *Lactobacillus *complex capsules for six months caused a more significant reduction in these OS markers than placebo capsules, indicating the remarkable positive influences of *Lactobacillus *in therapies for CRC by inhibition on OS. Similarly, Settanni et al. [37] discovered that *Lactobacilli *oral supplementation was effective in preventing CRC in animal models through the reduction of OS. The suppressive effects of *Lactobacillus *in OS and the underlying mechanisms are broadly studied. *Lactobacillus spp*. reverses the deoxynivalenol-induced morphological changes and oxidative stress on the intestines of broilers [38]. Three piglets-derived strains of *Lactobacillus*, including *Lactobacillus delbrueckii*, *Lactobacillus amylovorus*, and *Lactobacillus salivarius* exert an excellent anti-oxidant effect and intestinal barrier protection to reduce intestinal oxidative stress [39]. *Lactobacillus brevis* MTCC 1750 is a potent anti-oxidant source and extends life in Caenorhabditis elegans by the activation of the DAF-16 pathway [40].* Lactobacillus fermentum* U-21 exerts protective effects against paraquat-induced oxidative stress in Caenorhabditis elegans and mouse models [41]. *Lactobacillus plantarum* Y44 exerts protective effects against d-galactose-induced oxidative stress by remodelling gut microbiota and improving colonic barrier function in a mouse model of colon injury [42]. Interestingly, there is a high abundance of *Lactobacillus sakei* in people with obesity, and higher gut oxidative stress is associated with *Lactobacillus sakei* [43]. Thus, we made a hypothesis that certain *Lactobacillus *strains may stimulate cancers by inducing OS.

A previous study revealed that *Lactobacillus *could produce anti-oxidant enzymes to alleviate intestinal inflammation and oxidative stress, ameliorating inflammatory bowel disease by modulating the Nrf2 and MAPK signalling pathways [28]. ROS often triggers OS like hydrogen peroxide, superoxide anion, and hydroxyl radical, which serve as typical activators of the p38 MAPK and JNK pathways [44] [45]. ASK1, a mitogen-activated protein kinase kinase kinase in the JNK and p38 pathways, is activated in response to oxidative stress [46]. This study demonstrated the decline in ASK1 protein levels after *Lactobacillus *intervention. Mitogen-activated protein kinase kinases, such as MKK3 and MKK6, activate p38 in the p38 pathway and are activated by the same mitogenactivated protein kinase kinase kinases that function in the JNK pathway. Under conditions of oxidative stress, JNK and p38 are activated and induce the expression of the β-secretase gene [47]. Our study revealed that NRF2 and p38 pathways were suppressed, and the JNK pathway was not influenced after surgery in CRC patients. *Lactobacillus *complex capsules, by oral administration for six months, could decrease NRF2, p38, and JNK pathways. In line with our findings, it has been reported that *Lactobacillus rhamnosus *GG restrains the angiogenic potential of colorectal carcinoma cells by activating MAPK signalling [48]. Hiraishi et al. [49] proposed that *Lactobacillus plantarum* 06CC2 extract exerted a direct antitumour effect on colorectal cancer cells through the JNK/p38 MAPK signalling.

The limitation of the present study is that we did not perform a long-term clinical trial to observe the effects of *Lactobacillus* on the survival and tumour relapse of patients with CRC. Only three *Lactobacillus *strains, *Lactobacillus lactis*, *Lactobacillus acidophilus*, and *Streptococcus lactis* were studied, while the clinical effects of other strains need to be validated. The application of *Lactobacillus *complex capsules in clinical usage remains to be further verified in a larger cohort.

## Conclusion

This randomised controlled trial revealed that six-month oral supplementation with *Lactobacillus *complex capsules including *Lactobacillus lactis*, *Lactobacillus acidophilus*, and *Streptococcus lactis* twice daily can suppress expression of OS markers including CAT, GSH, GSSG, 8-oxodG, and F2-IsoPs and inhibit the NRF2, p38 MAPK, and JNK pathways in CRC patients after surgery. This study recommends the clinical usage of *Lactobacillus *in post-surgical CRC patients for a better prognosis.

## Dodatak

### Acknowledgements

No external funding was provided for this study.

### Conflict of interest statement

All the authors declare that they have no conflict of interest in this work.
